# Supplementary material for: Conservation assessment of human splice site annotation based on a 470-genome alignment
Source: Nucleic Acids Res. 2025 Mar 22;53(6):gkaf184. doi: 10.1093/nar/gkaf184 (PMC11928937; doi:10.1093/nar/gkaf184)
Supplement: gkaf184_Supplemental_File [file gkaf184_supplemental_file.pdf]

CONSERVATION ASSESSMENT OF HUMAN SPLICE SITE ANNOTATION BASED ON A 470-GENOME ALIGNMENT

SUPPLEMENTARY INFORMATION

Ilia Minkin
Department of Biomedical Engineering
Center for Computational Biology
Johns Hopkins University, Baltimore, MD 21211, USA
ivminkin@gmail.com

Steven L. Salzberg
Department of Biomedical Engineering
Center for Computational Biology
Department of Computer Science
Department of Biostatistics
Johns Hopkins University, Baltimore, MD 21211, USA
salzberg@jhu.edu

Chromosome	Aligned exons/genomes	Missing exons/genomes	Recovered exons/genomes
1	55,324,093	8,287,637	4,749,605
2	42,977,856	5,285,994	3,294,472
3	30,816,387	3,108,843	2,003,824

Supplementary Table S1: The number of aligned exons from the generated "Random" dataset in the original alignment of 470 mammals restricted to 405 species (column 2, defined as s_a in the main text), missing in the alignment (column 3, defined as s_m in the main text), and recovered using our synteny-based realignment procedure (column 4, defined s_r in the main text).

	Donor sites				Acceptor sites			
Organism	GENCODE$^{(*)}$	RefSeq$^{(*)}$	CHESS 3$^{(*)}$	Random	GENCODE$^{(*)}$	RefSeq$^{(*)}$	CHESS 3$^{(*)}$	Random
Protein-Coding								
Chimpanzee	88.07%	88.50%	87.92%	85.68%	87.61%	88.03%	87.21%	86.00%
Pygmy chimpanzee	87.80%	88.09%	87.47%	85.69%	87.15%	87.47%	86.61%	85.96%
Western lowland gorilla	84.44%	84.93%	83.91%	82.96%	84.02%	84.55%	82.96%	83.16%
Sumatran orangutan	80.09%	80.87%	80.34%	79.30%	81.47%	81.33%	80.32%	79.41%
Northern white-cheeked gibbon	76.75%	76.89%	76.13%	75.79%	78.32%	77.82%	76.91%	75.79%
Silvery gibbon	76.34%	76.16%	75.24%	75.48%	77.95%	77.11%	76.42%	75.55%
Crab-eating macaque	68.45%	69.37%	68.90%	68.57%	71.33%	72.44%	71.94%	68.57%
Rhesus monkey	68.52%	69.57%	69.07%	68.50%	71.36%	72.42%	71.91%	68.40%
Gelada	68.63%	69.22%	68.89%	68.53%	71.40%	71.79%	71.45%	68.47%
Japanese macaque	68.12%	68.76%	68.24%	67.97%	70.81%	71.57%	71.00%	67.99%
Golden snub-nosed monkey	68.13%	69.16%	68.99%	67.83%	70.69%	71.60%	71.24%	67.71%
Green monkey	68.34%	67.56%	68.46%	67.71%	70.41%	69.47%	70.54%	67.71%
Olive baboon	67.48%	67.62%	66.78%	67.56%	70.01%	70.15%	69.52%	67.58%
Mandrill	67.47%	67.95%	67.54%	67.14%	69.90%	70.30%	69.62%	67.12%
Ugandan red Colobus	67.24%	67.85%	67.29%	67.18%	69.52%	69.95%	69.33%	67.10%
Red shanked douc langur	67.34%	67.90%	67.54%	67.09%	69.78%	70.07%	69.64%	66.89%
Red guenon	67.31%	67.67%	67.16%	67.00%	69.67%	69.68%	68.90%	67.15%
Allen's swamp monkey	67.33%	67.65%	67.13%	66.91%	69.58%	69.89%	69.22%	66.86%
Francois's langur	67.36%	67.81%	67.31%	66.46%	69.96%	70.28%	69.94%	66.32%
Mona monkey	67.12%	67.33%	66.85%	66.68%	69.25%	69.32%	68.84%	66.64%
De Brazza's monkey	66.51%	66.76%	66.49%	66.35%	68.53%	69.12%	68.41%	66.35%
Hanuman langur	66.30%	66.81%	66.17%	66.05%	68.32%	68.57%	67.84%	65.98%
Proboscis monkey	50.59%	50.23%	50.26%	54.78%	53.07%	52.74%	52.23%	54.70%
White-faced saki	52.79%	52.75%	52.50%	52.67%	55.39%	56.22%	55.44%	52.72%
Black-handed spider monkey	52.55%	52.22%	52.49%	52.74%	54.82%	55.62%	54.86%	52.90%
Mantled howler monkey	52.17%	52.23%	52.16%	52.21%	54.16%	55.53%	54.53%	52.14%
Tufted capuchin	51.53%	51.47%	51.16%	52.17%	54.68%	54.91%	54.14%	52.33%
White-tufted-ear marmoset	51.41%	51.17%	50.84%	51.62%	54.22%	54.44%	53.80%	51.70%
Bolivian titi	51.11%	50.91%	50.70%	51.68%	54.17%	54.48%	53.59%	51.82%
White-fronted capuchin	50.56%	50.45%	50.00%	51.56%	53.51%	54.12%	53.03%	51.71%
lncRNA								
Chimpanzee	90.07%	89.73%	89.89%	-	89.73%	89.98%	89.59%	-
Pygmy chimpanzee	89.70%	89.51%	89.52%	-	89.48%	90.04%	89.43%	-
Western lowland gorilla	87.69%	87.14%	87.37%	-	87.60%	88.11%	87.45%	-
Sumatran orangutan	81.28%	81.50%	81.44%	-	83.30%	83.93%	83.62%	-
Northern white-cheeked gibbon	75.58%	75.80%	75.61%	-	77.34%	77.82%	77.28%	-
Silvery gibbon	74.88%	75.21%	74.91%	-	76.33%	77.18%	76.55%	-
Rhesus monkey	68.41%	68.21%	68.11%	-	71.37%	71.80%	71.10%	-
Crab-eating macaque	68.46%	68.24%	68.00%	-	71.34%	71.80%	71.05%	-
Gelada	68.23%	67.93%	67.87%	-	71.10%	71.65%	70.95%	-
Japanese macaque	68.00%	67.63%	67.66%	-	70.80%	71.14%	70.53%	-
Golden snub-nosed monkey	67.92%	67.63%	67.60%	-	70.60%	71.14%	70.46%	-
Green monkey	67.74%	66.42%	67.45%	-	70.77%	70.12%	70.37%	-
Red shanked douc langur	67.37%	66.94%	66.98%	-	70.12%	70.35%	69.97%	-
Olive baboon	67.36%	66.69%	66.83%	-	70.33%	70.40%	69.73%	-
Mandrill	67.12%	66.55%	66.62%	-	69.72%	70.47%	69.77%	-
Red guenon	66.80%	66.69%	66.68%	-	69.79%	70.30%	69.37%	-
Francois's langur	66.88%	66.38%	66.50%	-	69.69%	69.56%	69.13%	-
Allen's swamp monkey	66.43%	66.31%	66.10%	-	69.13%	69.75%	69.01%	-
Ugandan red Colobus	66.50%	66.07%	66.02%	-	69.17%	69.43%	68.68%	-
Mona monkey	66.24%	65.48%	65.64%	-	69.11%	69.39%	68.98%	-
De Brazza's monkey	65.81%	65.19%	65.26%	-	68.32%	68.89%	68.20%	-
Hanuman langur	65.74%	65.16%	65.26%	-	68.35%	68.26%	67.92%	-
Proboscis monkey	54.38%	52.97%	53.76%	-	56.79%	56.20%	55.88%	-
White-faced saki	51.23%	51.75%	51.46%	-	55.09%	55.98%	54.98%	-
Black-handed spider monkey	50.56%	50.82%	50.46%	-	54.75%	54.98%	54.18%	-
Tufted capuchin	50.25%	50.27%	50.04%	-	54.10%	54.60%	53.74%	-
Mantled howler monkey	50.15%	50.22%	49.91%	-	54.19%	54.19%	53.49%	-
White-fronted capuchin	49.71%	49.91%	49.47%	-	53.57%	53.78%	52.84%	-
Bolivian titi	49.45%	49.69%	49.38%	-	53.27%	53.89%	52.80%	-
Tamarin	48.89%	49.11%	49.00%	-	52.77%	53.27%	52.40%	-

Supplementary Table S2: Top 30 most common species in which donor and acceptor splice sites are conserved in different datasets, including the "Random" baseline dataset (for comparison). To compute these species, we only used splice sites from GENCODE, RefSeq, CHESS conserved in less than 50 species that appear to have a random-like conservation pattern. Each entry shows the proportion of splice sites that have their canonical dinucleotides conserved in certain species relative to the total number of sites in the respective category. The first half of the table shows sites from protein-coding genes, the second one shows sites from lncRNAs. For the second part, the "Random" column show dashes since the same set of generated sites were used as a baseline for comparing against both coding and non-coding genes.

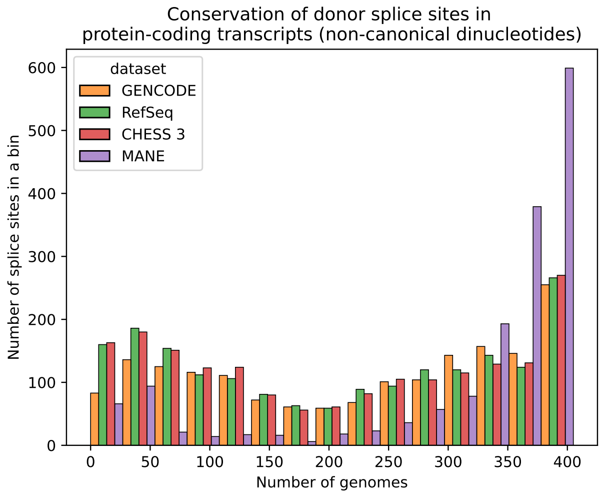

(a)

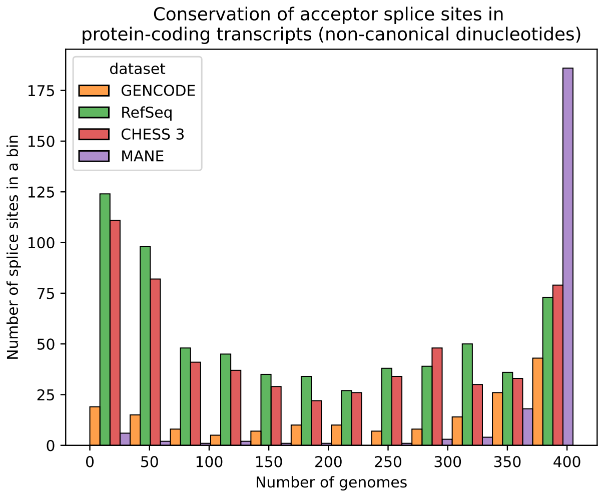

(b)

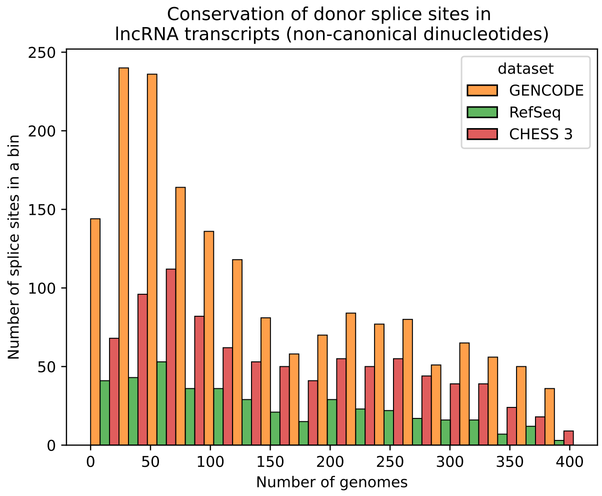

(c)

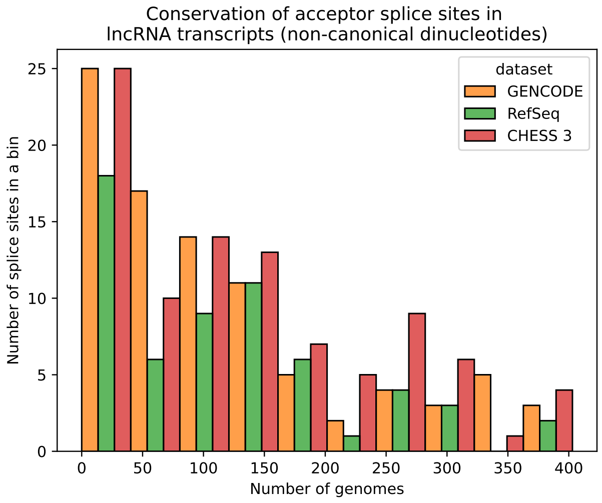

(d)

Supplementary Figure S1: The number of human splice sites with non-canonical dinucleotides in place of GT/AG conserved in 470 mammals, computed for donor (a) and acceptor (b) sites of protein-coding genes, and donor (c) and acceptor (d) sites of lncRNAs. Each point shows a number of splice sites conserved (y-axis) in a given number of species (x-axis), and data points are binned. For protein-coding genes, we created subsets GENCODE, RefSeq, and CHESS 3 from which we removed MANE annotations because each of these datasets is a superset of MANE; the resulting datasets are designated as GENCODE*, RefSeq*, and CHESS 3* correspondingly.

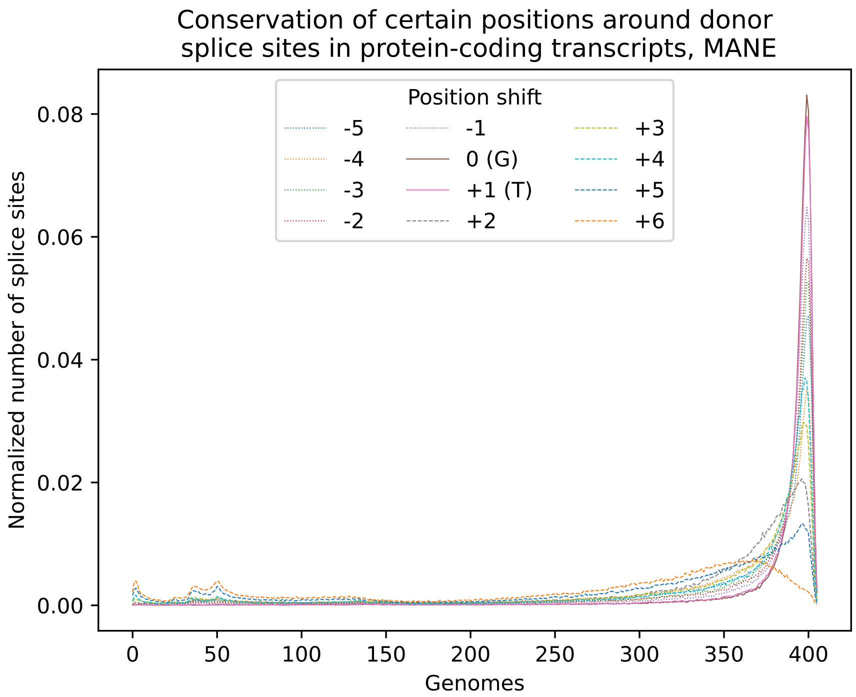

(a)

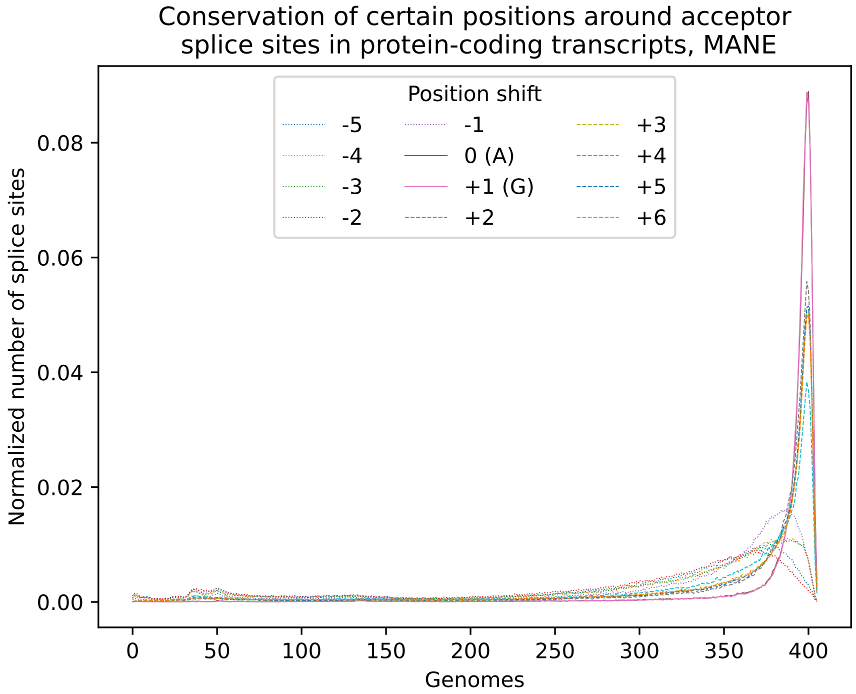

(b)

Supplementary Figure S2: Distribution of the number of donor (a) and acceptor (b) splice sites with a certain position around splice site motif conserved in a given number of species, for protein-coding genes from the MANE dataset. Each line represents the conservation of a position either down- or upstream of the "canonical" dinucleotides. For example, for donor splice sites 0 is usually "G", 1 is "T", and -1 is the first nucleotide upstream of the splice site. These nucleotides are shown in parentheses in the legends of the corresponding positions. Numbers are normalized by the total number of sites in the corresponding dataset in each category.

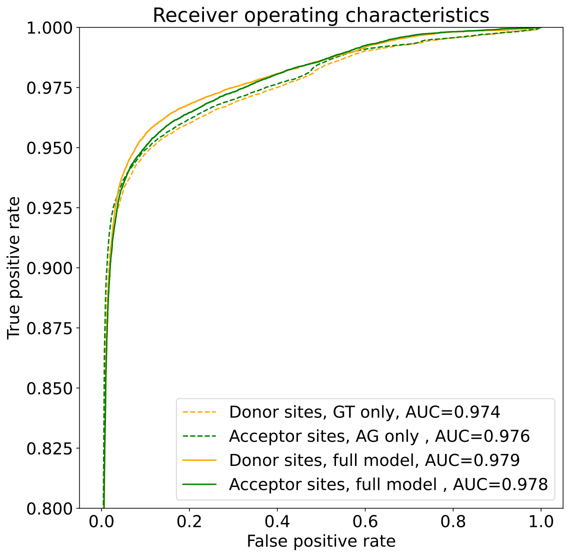

Supplementary Figure S3: Receiver operating characteristic (ROC) curve for logistic regression models trained to distinguish between GT/AG sites chosen at random from intronic sequences and splice sites from MANE. Dashed lines correspond to the models using only the conservation of the canonical dinucleotides themselves, while solid ones show the performance of the model taking into account the conservation of the positions in a larger window.

Model	Recall	Precision	F-score
Canonical dinucleotides only			
Donor sites	0.946	0.911	0.928
Acceptor sites	0.943	0.903	0.923
Full model			
Donor sites	0.943	0.923	0.933
Acceptor sites	0.949	0.949	0.949

Supplementary Table S3: Precision and recall of models classifying splice sites as either "well-supported" or "less-supported" on the test dataset containing. The first two rows show the performance of the models taking into account the canonical dinucleotides GT/AG only, while the next two rows correspond to the model considering a larger window. The classification threshold for probability is 0.5. Donor (acceptor) test set consisted of 36,000 false examples, randomly generated from intronic sequences, and 36,000 positive examples from MANE.

Dataset	All splice sites		“Well-supported” splice sites	
Protein-Coding	Donor	Acceptor	Donor	Acceptor
MANE	1,617	225	-	-
GENCODE*	1,737	172	556	83
RefSeq*	1,877	647	571	157
CHESS 3*	1,874	572	563	149
lncRNA				
MANE	-	-	-	-
GENCODE	1,746	89	169	8
RefSeq	419	60	28	3
CHESS 3	897	94	66	9

Supplementary Table S4: Summary statistics of splice site conservation analysis, non-canonical splice sites. The second and third columns represent the total number of donor and acceptor sites in each dataset. The third and fourth columns show the number of donor and acceptor splice sites classified as “well-supported” by our model.

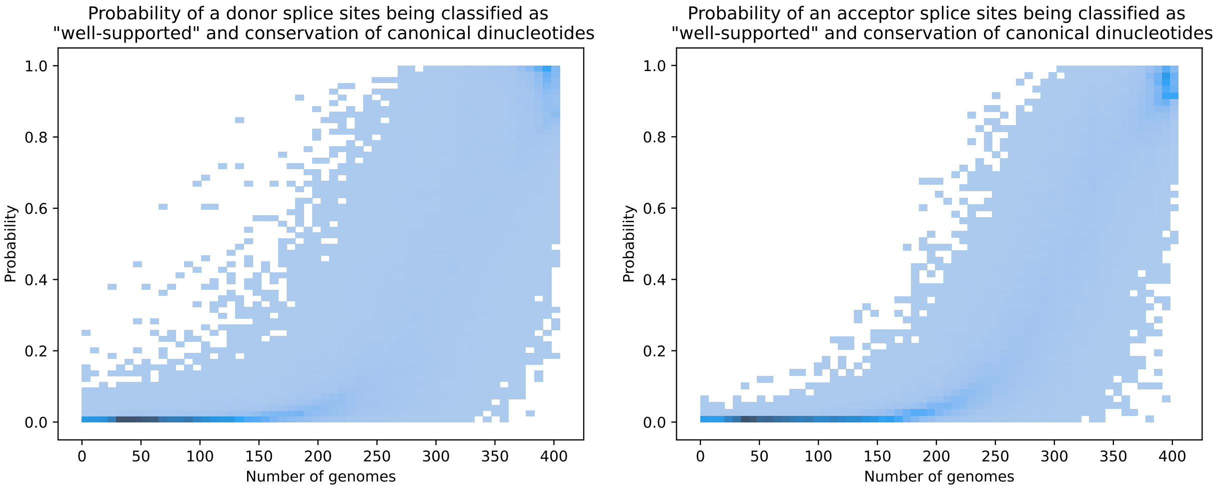

(a) (b)

Supplementary Figure S4: The relationship between the probability of a donor (a) and acceptor (b) splice sites classified as "well-supported" and the number of genomes in which the canonical dinucleotides are conserved. We used the "full model" that considers the conservation of the soliciting motif, as well as the canonical dinucleotides. Each panel depicts donor (acceptor) splice sites from protein-coding genes of GENCODE*, RefSeq*, and CHESS 3*, as well as form lncRNA genes of GENCODE, RefSeq, and CHESS 3.

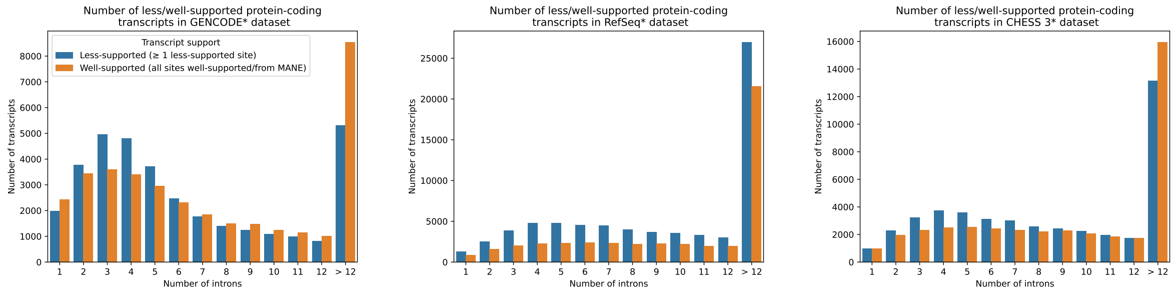

(a) (b) (c)

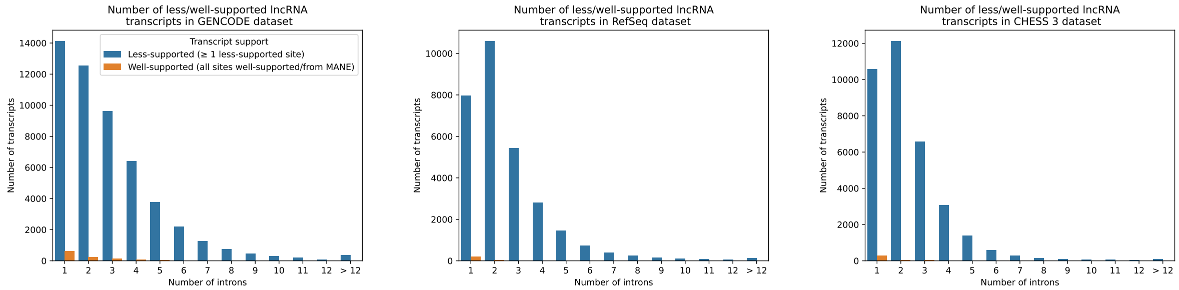

(d) (e) (f)

Supplementary Figure S5: The number of protein-coding and lncRNA transcripts in GENCODE, RefSeq, and CHESS 3 with a certain number of introns split by their support status. A transcript is defined as well-supported if all of its splice sites either are present in the MANE dataset or classified as "well-supported" by our model, otherwise the transcript is "less-supported. Panels (a)-(c) show transcripts from protein-coding genes, while (d)-(f) represent lncRNAs.

Category	Donor sites				Acceptor sites			
Inside a MANE exon	No		Yes		No		Yes	
"Well-supported"	No	Yes	No	Yes	No	Yes	No	Yes
GENCODE*	20,560	7,650	2,815	2,966	13,480	4,969	3,370	5,044
RefSeq*	32,813	24,988	2,510	2,549	25,315	21,741	2,859	4,245
CHESS 3*	23,958	21,170	2,667	2,679	19,566	18,330	2,988	4,575

Supplementary Table S5: Summary statistics of splice site conservation analysis, with sites being split by whether they are located inside a MANE exon or an intron. Each column shows either the number of "well-supported or "less-supported" splice sites from each dataset, located inside or outside MANE exons, in each dataset in both categories (donor and acceptor).

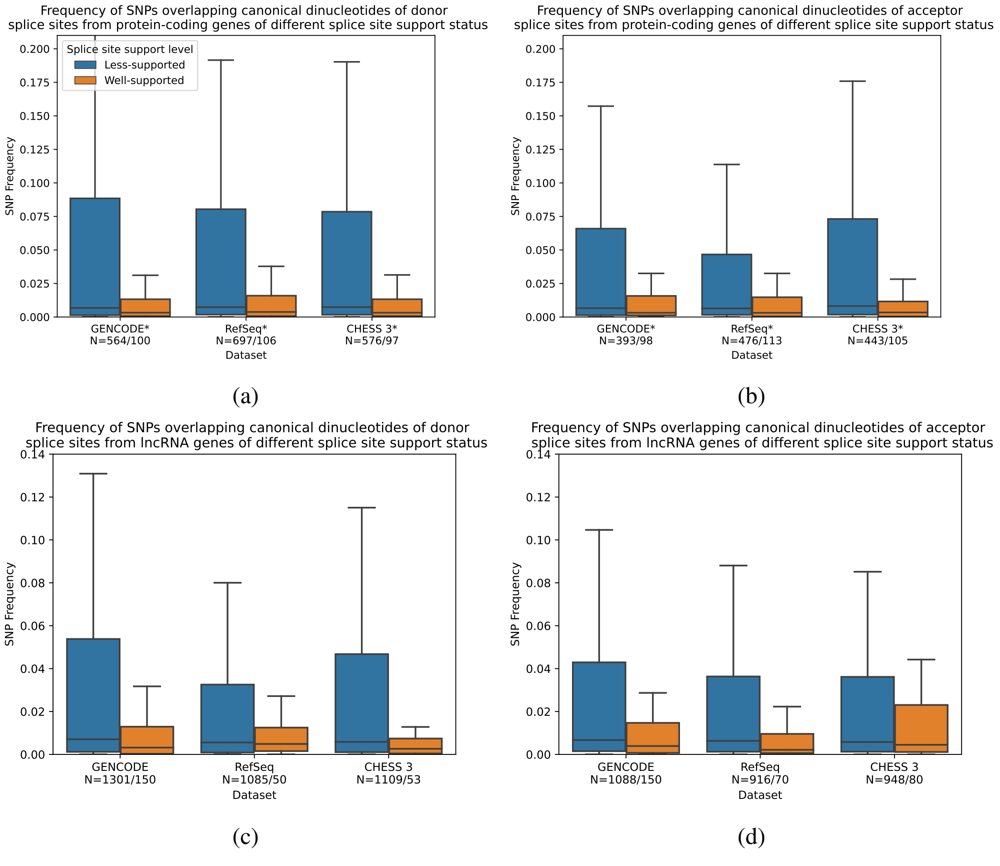

Supplementary Figure S6: Box plots showing the distribution of SNP frequencies from the gnomAD dataset overlapping the canonical dinucleotides GT/AG of splice sites of different support status as determined by our model. Panel (a) and (b) show donor and acceptor sites from protein-coding genes, while (c) and (d) illustrate donor and acceptor sites from lncRNAs. Each pair of boxplots shows the distribution of frequencies of splice sites from a particular dataset and support categories; numbers before the dataset title show the number of SNPs overlapping splice sites in well-supported/less-supported categories. Each box plot shows the median (solid horizontal line), the interquartile range (solid top and bottom borders of the box), and maximum values within 95% percentile (whiskers), outliers are not shown.

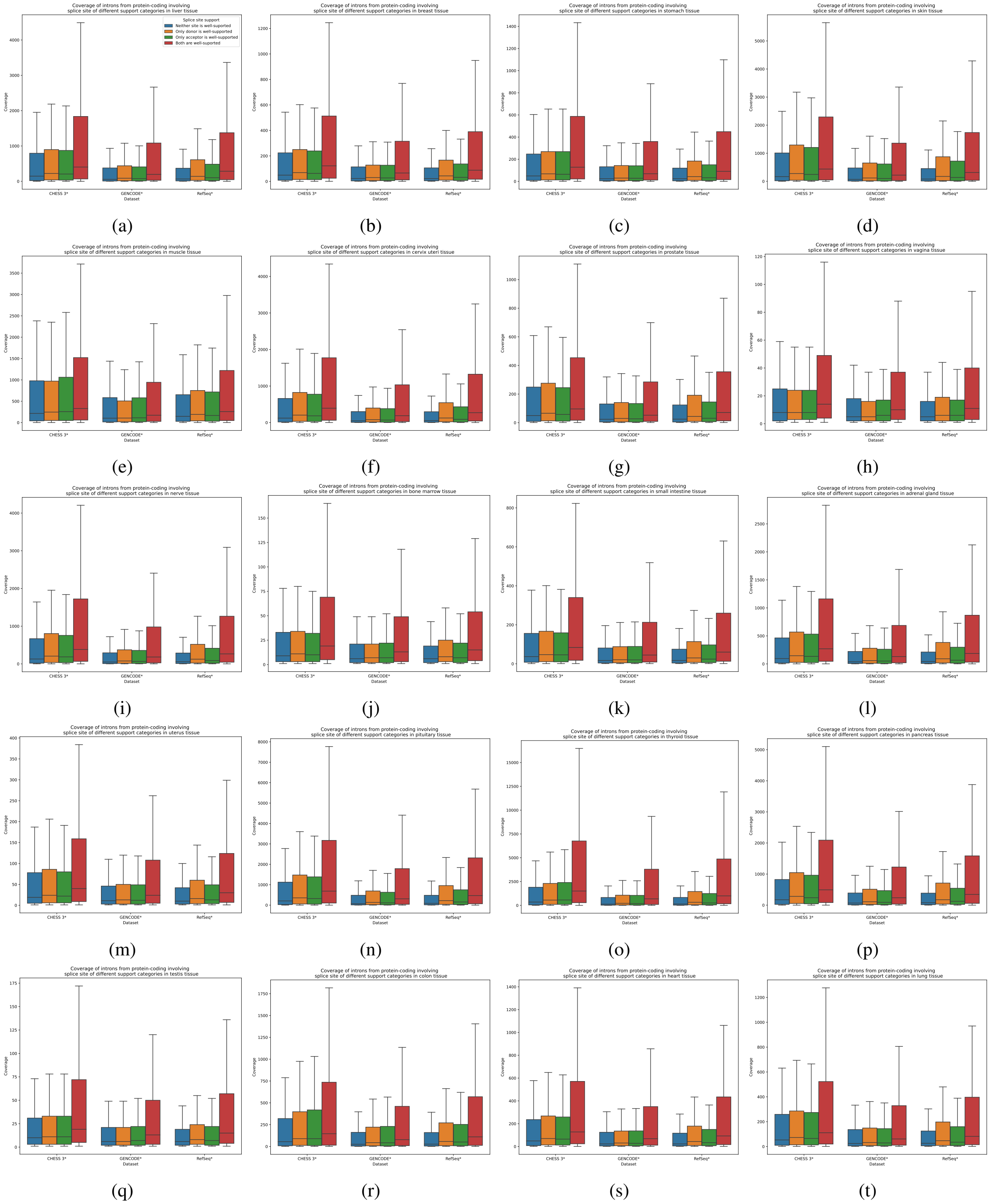

Supplementary Figure S7: Box plots showing coverage of introns from protein-coding genes of GENCODE*, RefSeq*, from CHESS 3* across different tissues. Each box plot shows the median (solid horizontal line), the interquartile range (solid top and bottom borders of the box), and minimum and maximum values within ±1.5 of the interquartile range (whiskers); outliers are not shown.

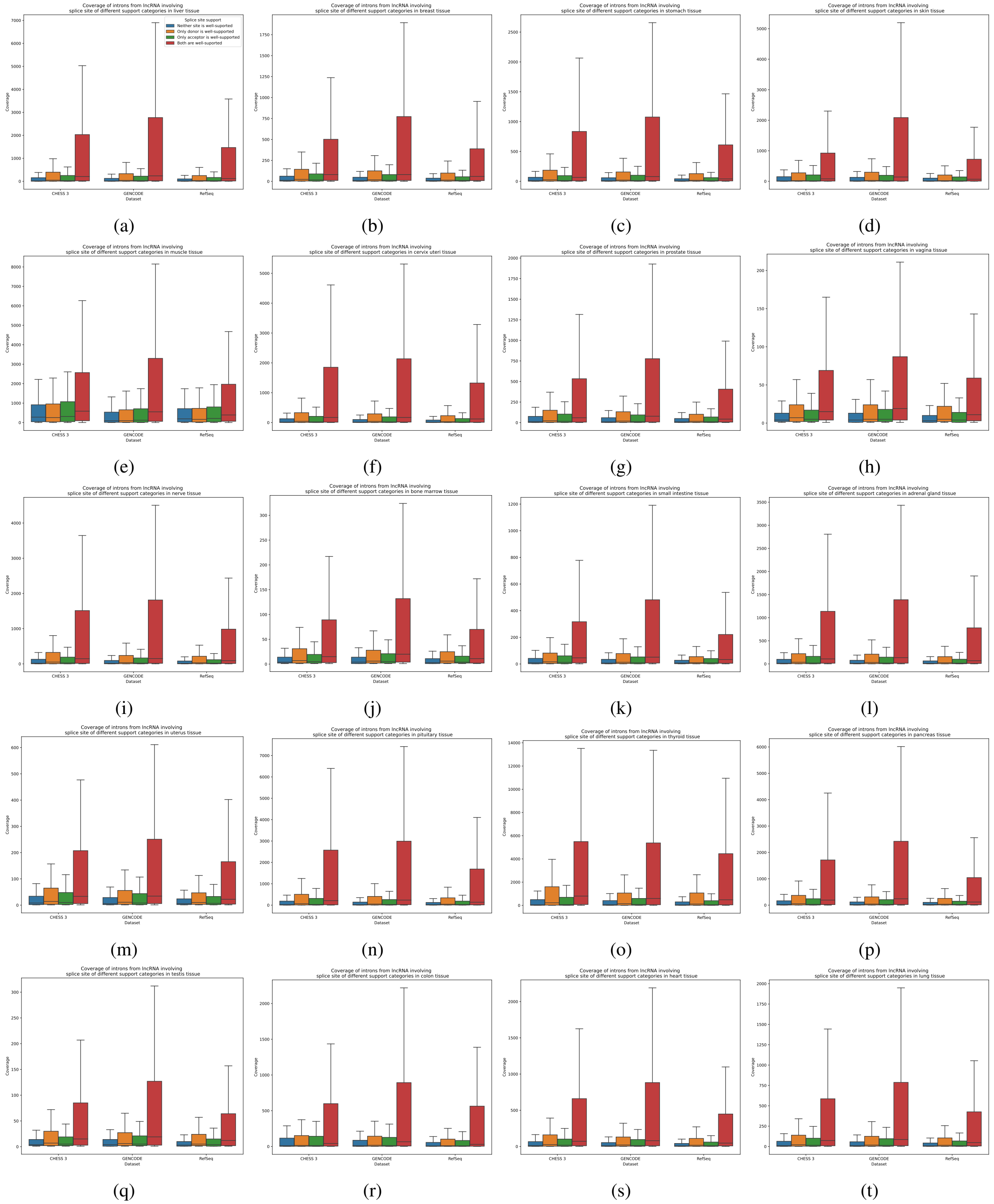

Supplementary Figure S8: Box plots showing coverage of introns from lncRNAs of GENCODE*, RefSeq*, from CHESS 3* across different tissues. Each box plot shows the median (solid horizontal line), the interquartile range (solid top and bottom borders of the box), and minimum and maximum values within ±1.5 of the interquartile range (whiskers); outliers are not shown.

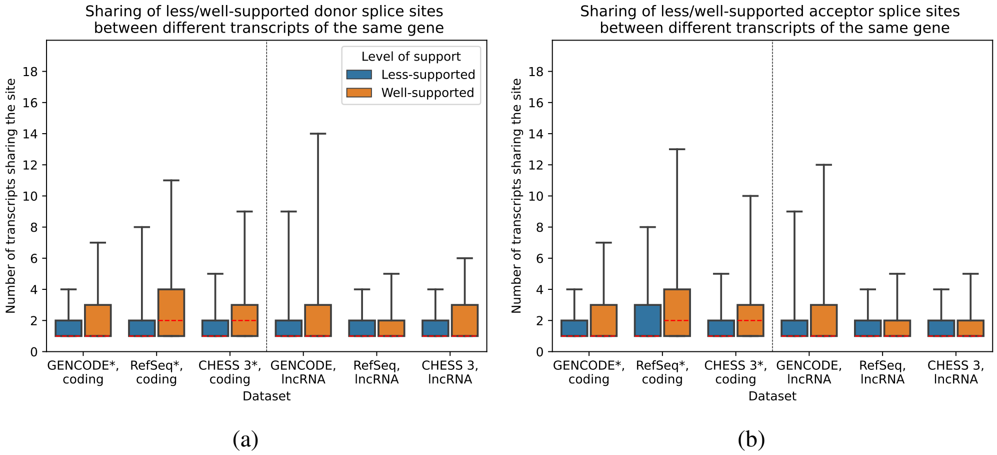

Supplementary Figure S9: Box plots showing the distribution of the number of transcripts sharing a certain donor (a) and acceptor (b) splice site, in GENCODE*, RefSeq*, and CHESS 3* datasets. The left part of each panel shows splice sites from protein-coding genes, while the right represents lncRNAs. Each box plot shows the median (dashed red line), the interquartile range (solid top and bottom borders of the box), and maximum values within 95% percentile (whiskers), outliers are not shown.

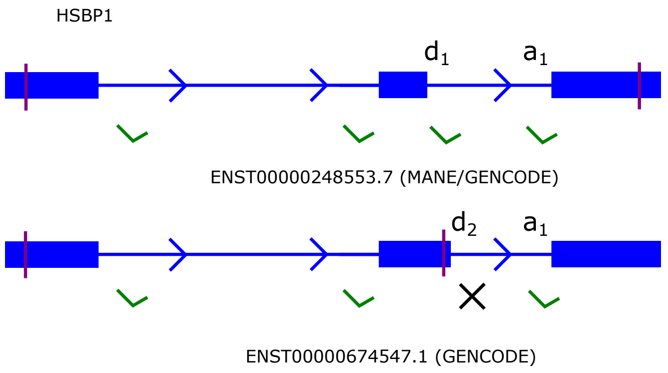

(a)

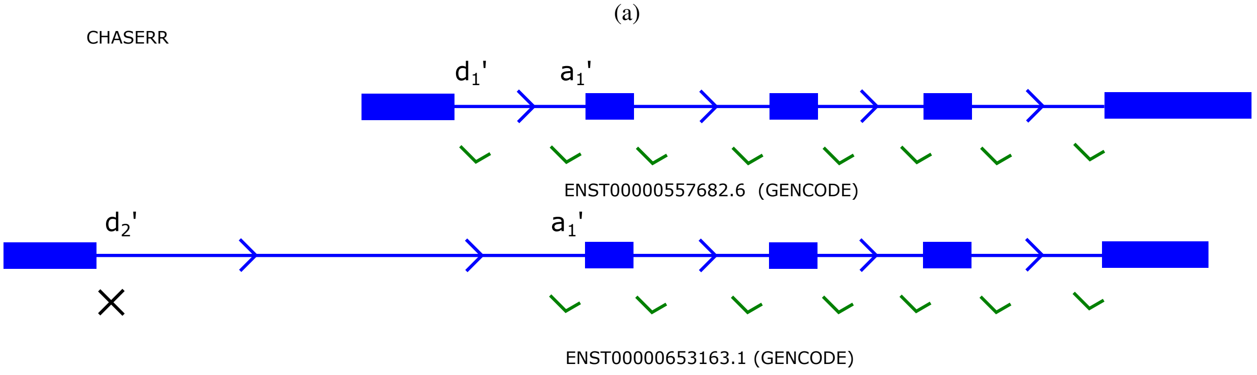

(b)

Supplementary Figure S10: This figure shows the structure of the transcripts containing less-supported splice sites that we believe could be misannotated. Solid blue boxes show exons, and blue arrows represent introns; green ticks indicate "well-supported" splice sites, while black crosses indicate “less-supported” ones, and vertical purple lines indicate start/stop codons. Figures are not drawn to an exact scale. Panel (a) shows two isoforms of the heat shock protein family B (small) member 1, or HSPB1 gene: MANE, ID ENST00000248553.7, upper transcript, and its alternative isoform from GENCODE, ID ENST00000674547.1, lower transcript that differs by a location of a donor splice site d_2. This alternative site results in a premature stop codon terminating the protein. The resulting protein is only 143 amino acids long. compared to the MANE isoform which contains 203 amino acids. Panel (b) shows two isoforms of lncRNA CHASERR from GENCODE: the upper isoform is ENST00000653163.1 and all its splice sites are “well-supported” according to our model. The lower isoform ENST00000557682.6 differs from the former transcript by the location of its first donor splice site (d'_2), which is less-supported. This isoform also occupies a genetic locus that is 4.7 times longer than its well-supported counterpart.

	Gene	HSBP1		CHASERR	
	Intron	d_1 (✓) a_1 (✓)	d_2(×) a_1 (✓)	d'_1 (✓) a'_1 (✓)	d'_2 (×) a'_1 (✓)
Tissue	Spleen	14,477,176	17	89,208	-
	Salivary Gland	13,461,418	5	123,743	-
	Blood	8,234,251	11	109,608	-
	Blood Vessel	5,605,781	12	32,065	-
	Ovary	5,154,826	8	29,174	-
	Thyroid	3,200,006	2	166,257	2
	Nerve	3,080,882	15	41,658	-
	Pituitary	2,829,673	-	79,553	-
	Breast	1,832,637	-	12,905	-
	Pancreas	1,576,185	-	54,498	-
	Liver	1,538,312	24	53,020	-
	Heart	1,214,850	-	11,977	-
	Skin	908,380	-	51,320	-
	Cervix Uteri	876,715	-	53,241	-
	Fallopian Tube	852,013	-	11,997	-
	Bladder	763,279	1	15,228	-
	Adrenal Gland	743,273	1	36,980	-
	Brain	680,486	-	14,915	-
	Lung	521,222	-	10,311	-
	Small Intestine	465,708	-	7,964	-
	Stomach	404,787	-	15,357	-
	Esophagus	362,020	-	9,530	-
	Kidney	309,843	-	13,341	-
	Adipose Tissue	303,877	-	5,161	-
	Colon	288,139	-	12,308	-
	Muscle	211,252	-	22,579	-
	Uterus	182,724	-	3,794	-
	Prostate	175,402	-	10,614	-
	Bone Marrow	90,179	-	1,011	-
	Testis	80,903	-	969	-
	Vagina	40,997	-	747	-

Supplementary Table S6: This table shows the aggregated number of reads across all samples in each tissue in the GTEx dataset supporting the exon junction described in the subsection “Case study: spotting potentially suspicious isoforms” of the main text. Columns 2 and 3 show the number of reads supporting introns (d_1, a_1) and (d_2, a_1) of the gene HSPB1, both sharing the same acceptor splice site; however, the donor site d_1 is well-supported, while the donor splice of d_2 is less-supported. Ticks indicate well-supported splice sites, while crosses show less-supported sites. Columns 4 and 5 depict the same date but for lncRNA gene CHASERR, and the support status is analogous: the donor site d'_1 is well-supported, while the donor splice of d'_2 is less-supported Supplementary Figure S10 shows the structure of these two genes and the locations of the splice sites inside each respective gene.
